# Beyond the Bone Marrow: A Case Report of Extramedullary Hematopoiesis in Advanced Primary Myelofibrosis

**DOI:** 10.7759/cureus.114220

**Published:** 2026-08-09

**Authors:** Duvan Alejandro Grisales Cano, William Almir Castellanos Olarte, Ana Maria Londoño Fonseca, Nazly Jullieth Bonilla Cardona, Sofia Salas Fernandez

**Affiliations:** 1 Department of Internal Medicine, Universidad Cooperativa de Colombia, Medellin, COL; 2 Department of Hematology, Universidad Nacional de Colombia, Medellin, COL; 3 Department of Medicine, Unidad Central Del Valle del Cauca, Tuluá, COL; 4 Department of Tropical Medicine, Universidad Corporación para Estudios en Salud (CES), Medellin, COL; 5 Department of Medicine, Universidad Cooperativa de Colombia, Medellin, COL

**Keywords:** extramedullary hematopoiesis, massive splenomegaly, myeloproliferative neoplasms, portal hypertension, primary myelofibrosis

## Abstract

Primary myelofibrosis (PMF) is a chronic myeloproliferative neoplasm characterized by progressive bone marrow fibrosis, cytopenias, splenomegaly, and extramedullary hematopoiesis (EMH). Although EMH most commonly involves the spleen and liver, extensive organ infiltration leading to portal hypertension and obstructive cholestasis is uncommon. We present the case of a 54-year-old man diagnosed with PMF who developed massive hepatosplenic EMH following prolonged interruptions to treatment caused by barriers to healthcare access. The disease progressed to an accelerated phase with extensive bone marrow fibrosis, grade 3 osteosclerosis, and 10.5% aberrant myeloid blasts without fulfilling diagnostic criteria for acute myeloid leukemia. Imaging demonstrated diffuse periportal infiltrative soft tissue causing biliary obstruction, massive splenomegaly, portal hypertension, and esophagogastric varices. The patient received hydroxyurea, ruxolitinib, and palliative hepatosplenic radiotherapy, achieving transient clinical and biochemical improvement. However, he subsequently developed radiation-induced bone marrow aplasia and died from progressive disease. This case underscores the aggressive clinical course of advanced PMF when continuous disease-modifying therapy is interrupted and highlights the importance of timely access to targeted treatment and a multidisciplinary approach for managing extensive EMH and its life-threatening complications.

## Introduction

Primary myelofibrosis (PMF) is a chronic, progressive myeloproliferative neoplasm (MPN) arising from clonal hematopoietic stem cells. It is characterized by bone marrow fibrosis, anemia, thrombocytopenia, splenomegaly, and constitutional symptoms [1,2]. Driver mutations involving Janus kinase 2 (JAK2), calreticulin (CALR), and the myeloproliferative leukemia virus oncogene (MPL), which encodes the thrombopoietin receptor, play a central role in the pathogenesis of PMF and other MPNs [3]. The diagnosis is established according to the World Health Organization (WHO) criteria, integrating bone marrow histopathology, molecular findings, clinical features, and the exclusion of other myeloid neoplasms [2].

As the disease progresses, ineffective bone marrow hematopoiesis promotes extramedullary hematopoiesis (EMH), which most commonly involves the spleen and liver, resulting in hepatosplenomegaly [2,4]. However, clinically significant hepatobiliary EMH is exceedingly uncommon. Most patients with hepatic involvement remain asymptomatic or present only with hepatomegaly. In contrast, obstructive jaundice caused by periportal EMH has been described only in isolated case reports and small case series [5-7]. In these rare cases, periportal infiltration by hematopoietic tissue may produce biliary obstruction, portal hypertension, and progressive liver dysfunction.

Current management of PMF is based on disease risk and symptom burden. It includes JAK inhibitors, particularly ruxolitinib; allogeneic hematopoietic stem cell transplantation for eligible patients; cytoreductive therapy; and low-dose radiotherapy for selected patients with symptomatic EMH or massive splenomegaly [2,3,8]. Despite these therapeutic advances, the optimal management of hepatobiliary EMH remains uncertain because of the limited number of reported cases.

We report an unusual case of advanced PMF presenting with massive hepatosplenic EMH associated with diffuse periportal infiltration, portal hypertension, obstructive cholestasis, and accelerated-phase disease. Unlike previously reported cases, the coexistence of these manifestations and the multidisciplinary management with ruxolitinib and hepatosplenic radiotherapy make this case a valuable contribution to the limited literature on this rare clinical presentation.

## Case presentation

A 54-year-old man with no significant personal or family medical history presented with a six-month history of colicky mesogastric abdominal pain associated with intermittent loose stools unrelated to dietary changes. He denied vomiting but reported marked asthenia, adynamia, and an unintentional 9-kg weight loss. On physical examination, his performance status was ECOG 2. He was deeply jaundiced and had marked abdominal distension with grade III ascites, positive fluid wave, and shifting dullness. The liver was palpable more than 5 cm below the right costal margin, and the spleen extended more than 8 cm below the left costal margin, consistent with massive hepatosplenomegaly. There was no clinically evident peripheral lymphadenopathy.

Initial laboratory findings are summarized in Table 1, demonstrating leukocytosis, anemia, thrombocytosis, and a leukoerythroblastic peripheral blood smear. Contrast-enhanced thoracoabdominal computed tomography (CT) demonstrated hepatosplenomegaly, with a liver measuring 20.5 cm and a spleen measuring 28.5 cm. Bone marrow biopsy revealed 70% cellularity, atypical megakaryocytic proliferation, and extensive fibrosis, consistent with a chronic MPN with morphological features of PMF. Polymerase chain reaction testing for BCR::ABL1 transcripts (p190, p210, and p230) was negative. Molecular testing for JAK2, CALR, and MPL mutations was not performed because these assays were unavailable at our institution at the time of diagnosis, and referral to another specialized center for molecular testing was not feasible due to healthcare system limitations. This represents a limitation of the case, as molecular characterization is currently recommended for the diagnosis and prognostic stratification of PMF.

**Table 1:** Laboratory findings during the clinical course ×10³/µL: thousands of cells per microliter, g/dL: grams per deciliter, %: percentage, mg/dL: milligrams per deciliter, U/L: units per liter

Laboratories	At diagnosis	At readmission (two years later)	Reference range
White blood cells	38.88	66.59	4.5-11.0 (10³/μL)
Hemoglobin	10.4	7.1	13.0-16.0 (gr/dL)
Hematocrit	31.2	21.6	38-50 (%)
Platelet count	680	944	150-450 (10³/μL)
Nucleated red blood cells in peripheral blood smear	4/100 leukocytes	2.5%	Absent
Blood urea nitrogen	18	44.39	6-20 (mg/dL)
Creatinine	0.8	0.97	0.7-1.3 (mg/dL)
Alkaline phosphatase	83	1249	44-147 (U/L)
Alanine aminotransferase	49	191	7-56 (U/L)
Aspartate aminotransferase	15	114	8-33 (U/L)
Total bilirubin	0.9	12.9	0.1-1.2 (mg/dL)
Direct bilirubin	0.2	9.17	0-0.3 (mg/dL)

Nevertheless, the diagnosis was established according to the WHO criteria based on clinical, hematologic, and bone marrow histopathological findings. The patient was classified as Dynamic International Prognostic Scoring System (DIPSS) intermediate-2 risk (3 points), and treatment was initiated with hydroxyurea and ruxolitinib. During follow-up, treatment was administered intermittently because of healthcare access barriers affecting medication availability. Although disease progression occurred during this period, this temporal association should not be interpreted as evidence of causality.

**Figure 1:**
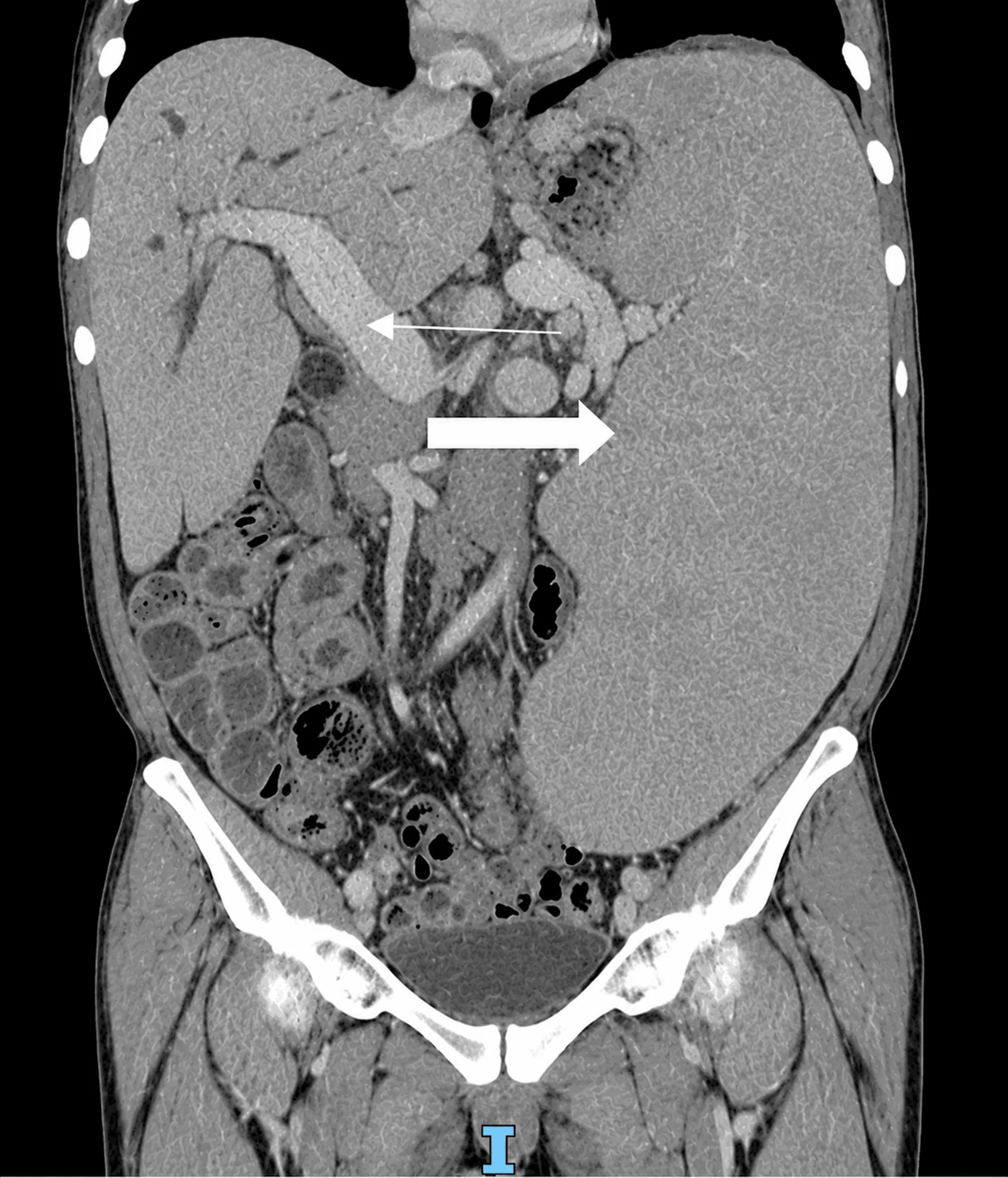
Contrast-enhanced abdominal CT Demonstrating hepatomegaly with signs of portal hypertension (thin white arrow) and massive splenomegaly extending from the left upper quadrant to the left iliac fossa, with an estimated splenic volume of 6,100 mL (thick white arrow). CT: computed tomography

Two years later, the patient was readmitted because of upper gastrointestinal bleeding secondary to esophageal and gastric varices requiring endoscopic band ligation. He also developed progressive jaundice and cholestatic abdominal pain. Laboratory findings at readmission are summarized in Table 1, demonstrating worsening hematologic abnormalities together with a marked cholestatic pattern of liver injury. Abdominal ultrasound showed intrahepatic biliary duct dilatation and cholestasis without evidence of cholecystitis. Contrast-enhanced CT of the abdomen demonstrated hepatomegaly measuring 22.8 cm, a patent but markedly dilated extrahepatic portal vein (28 mm), mild diffuse intrahepatic biliary dilatation, cholelithiasis, and massive splenomegaly measuring 25.6 × 13.2 × 34.7 cm, with an estimated splenic volume of 6,100 mL (Figure 1). Heterogeneous enhancement of the inferolateral splenic parenchyma was observed without focal lesions or splenic infarction.

Magnetic resonance cholangiopancreatography demonstrated extensive infiltrative periportal soft tissue with restricted diffusion, progressive narrowing, and focal occlusion of the intrahepatic bile ducts, resulting in upstream biliary dilatation. The spleen measured 36 cm in maximum longitudinal diameter and contained multiple solid nodules ranging from 0.8 to 3.5 cm, with associated mass effect on the gastrointestinal tract, pancreas, and retroperitoneal vascular structures. Based on these findings, the differential diagnosis included cholangiocarcinoma, lymphoma, metastatic disease, leukemic infiltration, IgG4-related disease, and infectious infiltrative disorders. These entities were excluded through correlation of the clinical presentation, laboratory evaluation, cross-sectional imaging, and bone marrow histopathology, which collectively supported hepatobiliary EMH as the most likely diagnosis.

A repeat bone marrow biopsy with flow cytometry demonstrated granulocytic proliferation with 10.5% aberrant myeloid blasts, consistent with accelerated-phase PMF, without fulfilling diagnostic criteria for acute myeloid leukemia. Histopathological examination also demonstrated grade 3 osteosclerosis, extensive fibrosis, and marked megakaryocytic dysplasia. Based on these findings, the patient was reclassified as high risk (DIPSS score = 5).

Treatment with hydroxyurea and ruxolitinib was restarted and adjusted according to the patient's clinical status in accordance with contemporary international guidelines and standard clinical practice for PMF. Palliative hepatosplenic radiotherapy was subsequently administered as adjunctive therapy, consisting of 2 Gy delivered in 10 fractions of 0.2 Gy over two weeks. Hydroxyurea was temporarily discontinued during radiotherapy to minimize the risk of severe treatment-related cytopenias. The patient experienced transient clinical and biochemical improvement, including a reduction in total bilirubin to 7.45 mg/dL and direct bilirubin to 5.96 mg/dL. Subsequently, he developed bone marrow aplasia requiring transfusion support and treatment with pegylated granulocyte colony-stimulating factor. Despite supportive management, his clinical condition progressively deteriorated, and he died as a consequence of advanced PMF.

## Discussion

EMH is an adaptive process in which hematopoietic progenitor cells proliferate outside the bone marrow in response to ineffective hematopoiesis or progressive bone marrow failure, most commonly in patients with chronic MPNs [5,6]. Although the spleen and liver are the most frequent sites of involvement, clinically significant hepatobiliary EMH remains exceptionally uncommon. Most reported cases present with hepatomegaly, portal hypertension, or abdominal pain, whereas diffuse periportal infiltration resulting in obstructive cholestasis and jaundice has been described only in isolated case reports [7-11]. Consequently, the coexistence of massive hepatosplenic EMH, portal hypertension, obstructive cholestasis, and accelerated-phase PMF represents an exceptionally rare clinical presentation.

This patient developed three major complications that reflected progressive disease: portal hypertension, obstructive cholestasis, and accelerated-phase PMF. Each of these findings was supported by concordant clinical, laboratory, radiologic, and pathologic evidence. Portal hypertension was clinically manifested by esophagogastric variceal bleeding. It was accompanied by marked splenomegaly on CT, portal vein dilation measuring 28 mm, and massive splenic enlargement exceeding 6 L in volume. Obstructive cholestasis was reflected by progressive jaundice, marked elevations in total and direct bilirubin and alkaline phosphatase, together with magnetic resonance cholangiopancreatography demonstrating diffuse periportal infiltrative soft tissue causing progressive narrowing and obstruction of the intrahepatic bile ducts. Finally, progression to accelerated-phase disease was confirmed by repeat bone marrow biopsy demonstrating grade 3 fibrosis, osteosclerosis, megakaryocytic dysplasia, and 10.5% aberrant myeloid blasts on flow cytometry.

Several mechanisms have been proposed to explain hepatobiliary involvement in PMF. Infiltration of periportal tissues by hematopoietic cells may produce extrinsic compression of the biliary tree. In contrast, intrahepatic sinusoidal infiltration and platelet-rich microthrombi may contribute to portal hypertension, cholestatic liver injury, and hepatic dysfunction independently of JAK2 mutational status [12,13]. Likewise, splenic fibrosis has been associated with increased megakaryocyte accumulation, supporting the concept that megakaryocytes actively contribute to both fibrosis and EMH progression [12]. The constellation of imaging findings and biochemical abnormalities observed in our patient is consistent with these proposed pathophysiological mechanisms.

Although hepatobiliary EMH has been reported previously, published cases remain scarce and generally describe isolated manifestations such as obstructive jaundice, portal hypertension, or focal hepatic masses. In contrast, our patient presented with simultaneous massive hepatosplenic EMH, diffuse periportal infiltration producing obstructive cholestasis, clinically significant portal hypertension with variceal bleeding, and accelerated-phase PMF. To our knowledge, few published reports have documented this combination of advanced clinical manifestations occurring concurrently, emphasizing the educational value of this case and expanding the spectrum of severe hepatobiliary complications associated with PMF.

Accelerated-phase PMF represents a critical stage in disease evolution. According to both the 2022 WHO classification and the International Consensus Classification, accelerated-phase disease is defined by the presence of 10-19% blasts in peripheral blood or bone marrow, whereas 20% or more blasts establish transformation to acute myeloid leukemia [7]. Patients entering the accelerated phase experience substantially reduced survival and a markedly increased risk of leukemic transformation compared with those in the chronic phase. In our patient, the identification of 10.5% aberrant myeloid blasts, together with worsening bone marrow fibrosis and osteosclerosis, fulfilled diagnostic criteria for accelerated-phase PMF without transformation to acute myeloid leukemia, providing a pathological explanation for the rapid clinical deterioration and poor outcome.

Splenomegaly is a major prognostic feature in PMF. Progressive splenic enlargement reflects increasing EMH, disease burden, and neoangiogenesis, all of which have been associated with inferior survival [14]. CT-derived spleen volume has emerged as an objective prognostic marker, with larger splenic volumes correlating with shorter leukemia-free and overall survival. In the present case, the spleen measured approximately 6,100 mL, illustrating the extreme degree of extramedullary disease and supporting its association with advanced disease biology.

Current management of intermediate-2 and high-risk PMF includes JAK inhibitors, particularly ruxolitinib, which remains the standard first-line therapy for symptomatic splenomegaly and constitutional symptoms. At the same time, allogeneic hematopoietic stem cell transplantation is the only potentially curative treatment for eligible patients. Cytoreductive therapy with hydroxyurea may be added in patients with persistent leukocytosis or thrombocytosis. The COMFORT-I and COMFORT-II trials demonstrated significant reductions in spleen volume and symptom burden and improved overall survival among patients receiving ruxolitinib compared with conventional therapy or placebo [15]. More recently, combination therapy with hydroxyurea and ruxolitinib has shown improved hematologic control in patients with persistent proliferative disease without a significant increase in cumulative toxicity [16]. In our patient, hydroxyurea and ruxolitinib were administered according to contemporary international recommendations for high-risk PMF; however, treatment continuity was limited by healthcare access barriers. Although disease progression occurred during this period, a causal relationship cannot be established from a single case report.

Splenectomy has historically been considered for patients with symptomatic massive splenomegaly, refractory transfusion requirements, or compressive symptoms. However, its use has declined because of substantial perioperative morbidity and mortality and the availability of effective JAK inhibitors capable of reducing spleen size without surgical intervention [17,18].

Radiotherapy remains an important palliative option for symptomatic EMH involving the spleen or liver when rapid local disease control is required, or systemic therapy alone is insufficient. Because hematopoietic tissue is highly radiosensitive, low-dose irradiation (generally 0.5-3 Gy delivered over multiple fractions) is usually preferred to achieve symptomatic improvement while minimizing hematologic toxicity. In the Mayo Clinic experience, 94% of patients undergoing splenic irradiation achieved meaningful reduction in spleen size, although treatment-related cytopenias occurred in approximately 44% of cases [19]. Similarly, hepatic irradiation has been associated with reductions in hepatomegaly and symptomatic improvement in selected patients with hepatobiliary EMH [20]. Reports involving accelerated-phase PMF have also described rapid reductions in splenic size and leukocyte counts using very low-dose splenic irradiation protocols. However, prolonged cytopenias remain the principal dose-limiting toxicity [19]. In the present case, hepatosplenic radiotherapy was selected as a palliative strategy because of symptomatic massive hepatosplenomegaly, portal hypertension, and biliary obstruction. Hydroxyurea was temporarily discontinued to reduce the risk of severe myelosuppression. Although transient biochemical improvement was observed, including declining bilirubin concentrations, the patient subsequently developed bone marrow aplasia, a recognized complication in individuals with markedly impaired baseline hematopoietic reserve.

This case highlights several clinically relevant lessons. First, hepatobiliary EMH should be considered in patients with PMF who develop cholestatic liver injury, portal hypertension, or obstructive jaundice without an alternative explanation. Second, integration of laboratory findings, cross-sectional imaging, and bone marrow pathology is essential to distinguish EMH from other infiltrative conditions, including lymphoma, cholangiocarcinoma, metastatic disease, IgG4-related disease, and infectious hepatic processes. Finally, this report expands the limited literature describing extensive hepatobiliary EMH complicated by accelerated-phase PMF. It underscores the importance of multidisciplinary management, timely access to disease-modifying therapy, and individualized palliative strategies in patients with advanced disease.

## Conclusions

PMF remains a clinically challenging MPN because of its heterogeneous presentation, progressive course, and potential to develop life-threatening complications such as extensive EMH. This case illustrates a rare presentation of advanced PMF with massive hepatosplenic EMH resulting in portal hypertension, obstructive cholestasis, and accelerated-phase disease. Although the patient's disease progression occurred during a period of interrupted treatment, the aggressive natural history of high-risk PMF likely also contributed to the unfavorable outcome. Therefore, this case should be interpreted as suggesting, rather than establishing, the potential clinical consequences of limited access to continuous disease-modifying therapy.

The patient's transient response to combined treatment with hydroxyurea, ruxolitinib, and hepatosplenic radiotherapy supports the role of individualized multimodal management in selected patients with advanced symptomatic disease; however, no conclusions regarding treatment efficacy can be drawn from a single case. This report also has important limitations, including the unavailability of molecular characterization (JAK2, CALR, and MPL mutation testing), which is currently recommended for diagnostic confirmation and prognostic stratification, and the inherent inability of an individual case report to establish causality or generalize therapeutic outcomes. Nevertheless, this case expands the limited literature on hepatobiliary EMH in PMF. It highlights the importance of integrating clinical, laboratory, imaging, and pathological findings to facilitate early recognition and multidisciplinary management of this rare but potentially fatal complication.
